# Fix the Flat: Applying the Principles of Tire Aerosol Repair to Prevent Exsanguination From a Hemorrhagic Intrahepatic Mass

**DOI:** 10.7759/cureus.19530

**Published:** 2021-11-13

**Authors:** Chase Tenewitz, Taylor S Harmon, Mika Matteo, Sanjay Lamsal, Jerry Matteo

**Affiliations:** 1 Radiology, Mercer University School of Medicine, Savannah, USA; 2 Radiology, University of Florida College of Medicine, Jacksonville, USA; 3 Radiology, University of Florida, Gainesville, USA

**Keywords:** percutaneous intervention, amplatzer occluder, amplatzer septal occluder, fix-a-flat, fluoroscopy, hemorrhage, enterohepatic fistula, abscessogram, interventional radiology, amplatzer plug device

## Abstract

Metastatic neoplasias often require increased blood supply for proliferation. Tumors that outgrow their blood supply can undergo necrosis, leading to the formation of mass-like abscesses. Depending on the location, these lesions can form fistulas with nearby organs resulting in poor patient outcomes. Interventional operators may use unconventional methods to the benefit of patients when resolving these complex lesions. The following case describes a patient with a large right hemorrhagic intrahepatic collection and formation of a duodenal fistula, resulting in acute blood loss anemia. Although there is not a standardized treatment for this complication, we present a novel therapeutic technique that incorporates similar principles analogous to the standard canned aerosol tire repair device.

## Introduction

Hepatic abscesses are circumscribed, necrotic collections of inflammatory tissue that can develop as a result of infectious intra-abdominal agents or injury [1]. Although occurring less frequently, hepatic abscesses can form secondarily to the necrosis of intrahepatic metastases [2]. Without proper treatment, intrahepatic abscesses can rupture, develop pleuropulmonary complications, and even embolize or form gastroenteric fistulas [2-3]. Hepatic abscess fistulization with various segments of the gastrointestinal tract, including the duodenum, stomach, hepatic flexure of the large intestine, or ascending colon, occurs significantly less frequently compared to other intrahepatic abscess complications [4-7].

Initial diagnosis of a hepatoduodenal fistula can be seen in a contrast-enhanced computed tomography (CT) scan of the abdomen followed with a confirmatory fistulogram [4-7]. Currently, there are no established treatment guidelines for hepatoduodenal fistulas. Treatment varies from conservative antibiotic therapy and catheter drainage to invasive surgical resection [4-7]. Although conservative management with antibiotics and catheter drainage provides symptomatic relief of hepatoduodenal fistulas, the patient must tolerate the inconvenience of an intrahepatic drain. Furthermore, intrahepatic drain placement may never completely resolve either the intrahepatic abscess or fistula. Resolution of a hepatoduodenal fistula with a more invasive approach, such as surgical resection, provides a benefit over percutaneous intervention. However, the majority of these patients are typically poor surgical candidates and would have increased recovery times or generally poor outcomes. The location of the necrotic tumor or abscess is an important factor to consider when choosing how to intervene.

To provide both a conservative and minimally invasive method for definitive treatment of a hemorrhagic liver abscess, the principles behind the standard canned aerosol tire repair device are used. Commercially available aerosol tire repair is commonly used to repair a flat automobile tire by releasing a liquid tire matrix to seal a leak from the inside while simultaneously filling the tire with air [8]. The following case demonstrates an analogous percutaneous method for repairing a hemorrhagic necrotic intrahepatic abscess which actively exsanguinates into the bowel.

## Technical report

**Figure 1:**
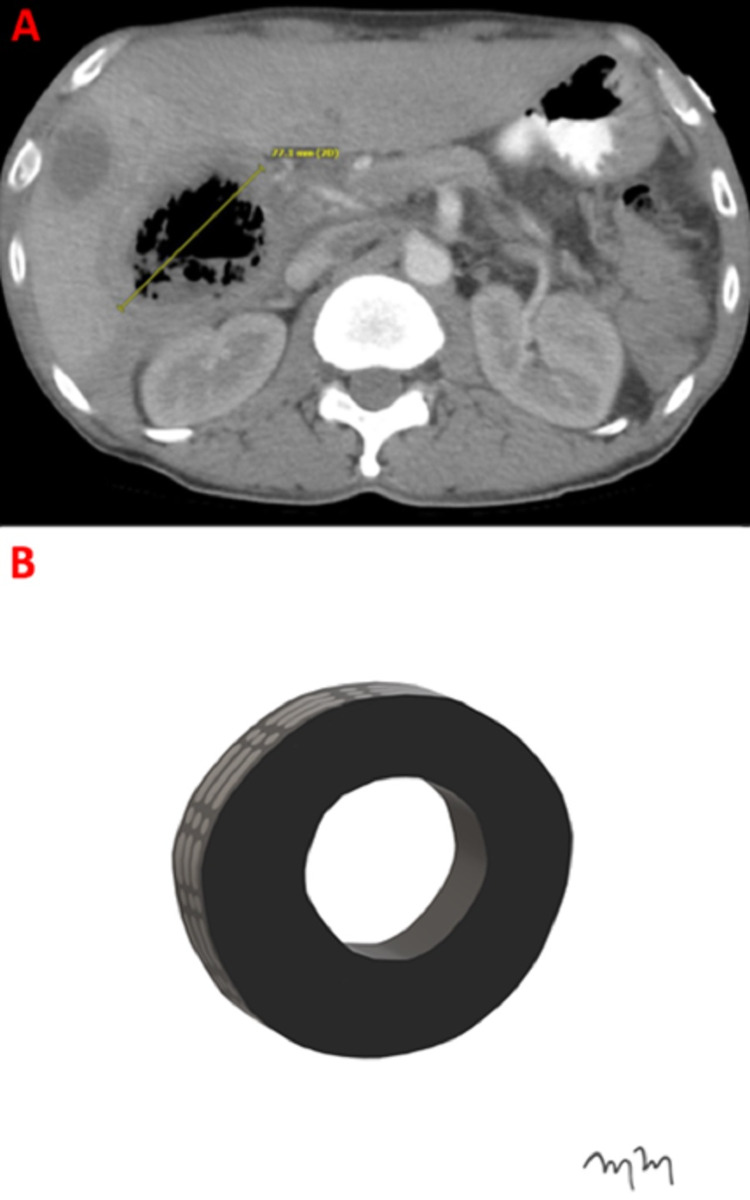
Intrahepatic Abscess Representing an Inflated Tire An axial computed tomography with intravenous contrast at the level of the inferior right hepatic lobe demonstrates a 77.1-millimeter abscess within hepatic segment six (A). An artist rendition image shows an inflated tire, representing the intrahepatic abscess (B).

A 57-year-old male with a past medical history of a right hepatic lobe metastatic squamous cell carcinoma and intrahepatic abscess presents for percutaneous management. The patient had a normal white blood cell count and was afebrile. A contrast-enhanced CT shows the right hepatic lesion (Figure 1).

**Figure 2:**
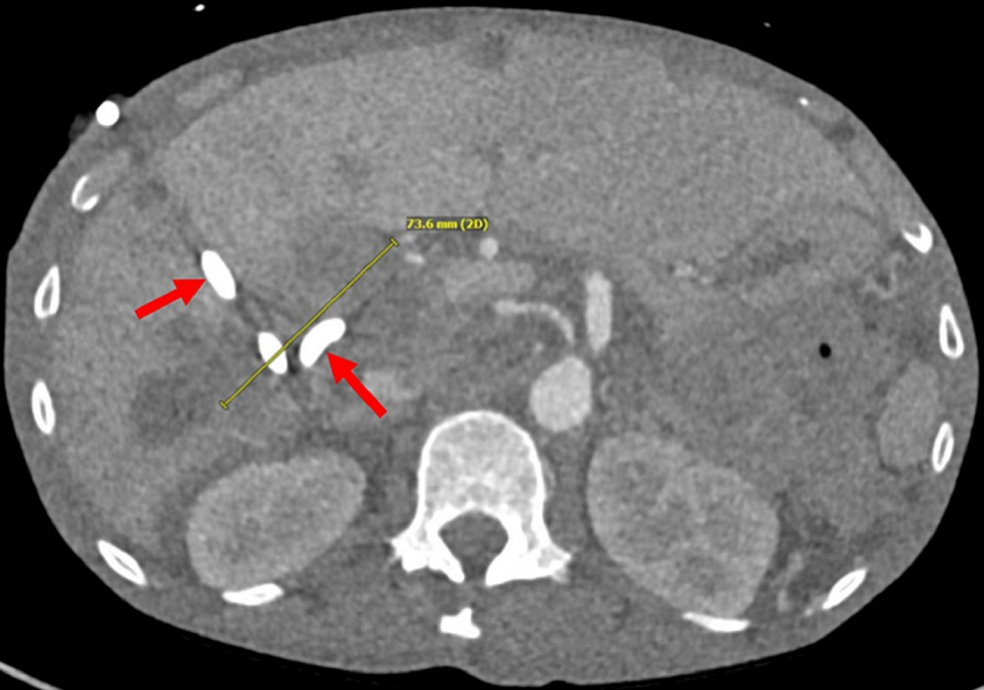
Pigtail Catheter Placed Within the Intrahepatic Abscess A post-procedural axial computed tomography at the level of the inferior right hepatic lobe demonstrates the percutaneous 10 French pigtail drainage catheter (red arrows) centered within the intrahepatic abscess (yellow measure).

A CT guided pigtail drain was placed inside the abscess (Figure 2).

After multiple drain exchanges, it was noted that the hepatic mass continued to grow and the abscess cavity fistulized to the duodenum. A computed tomography angiography excluded any active extravasation from the intrahepatic arteries or branches of the celiac axis, such as the gastroduodenal artery. The patient then developed fatigue and drainage of bright red blood from the liver abscess requiring multiple blood transfusions. The patient was not a surgical candidate based on the metastatic spread of the disease and the location of the large necrotic mass. The plan was for chemotherapy; however, the patient needed definitive and urgent treatment to combat the friable tissue from further hemorrhage. Interventional radiology was reconsulted for percutaneous hemostasis.

**Figure 3:**
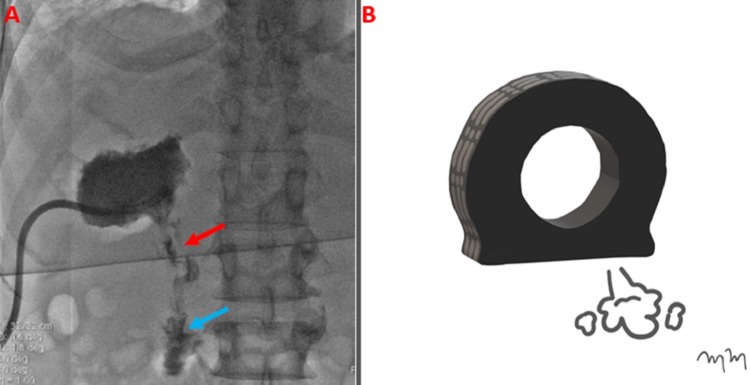
Actively Deflating Tire Representing Hemorrhagic Intrahepatic Abscess A contrast injected fluoroscopic guided drainage catheter interrogation (abscessogram) reveals an enterohepatic fistulous connection (red arrow) extending into the duodenum (blue arrow) (A). An artist rendition image shows an actively deflating tire, representing the hemorrhagic intrahepatic abscess with active bleeding through the fistulous connection (B).

The patient was brought to the angiosuite, where the right upper quadrant intrahepatic percutaneous drain was anesthetized with lidocaine. A hand contrast injection was performed through the existing percutaneous drainage catheter, which revealed the catheter to be within the appropriate position. The intrahepatic abscess cavity was relatively unchanged in size, and there was a persistent hepatoduodenal fistula (Figure 3).

**Figure 4:**
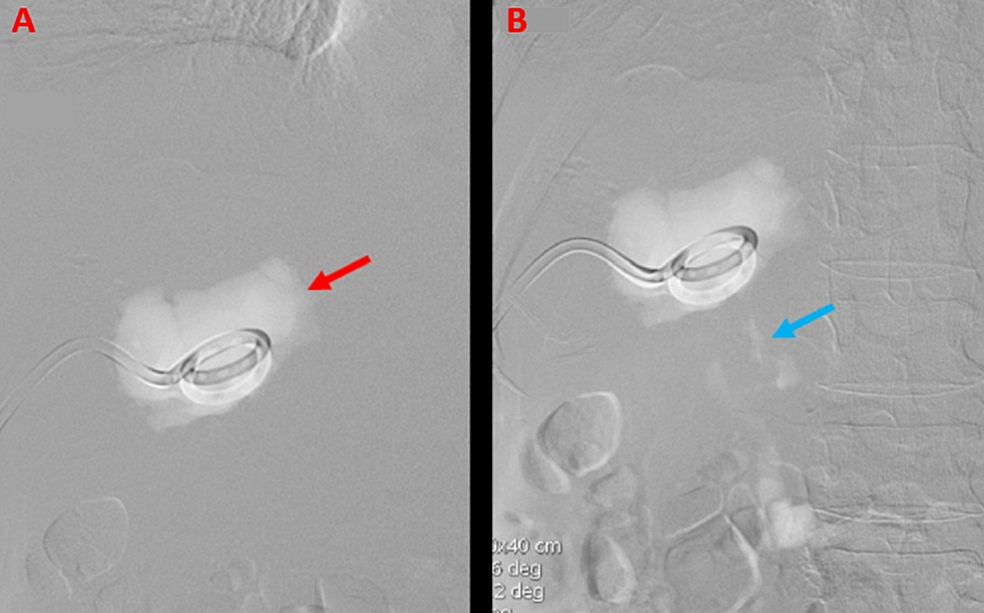
Intrahepatic Abscess Confirmed by Carbon Dioxide Injection A same day intra-procedural abscessogram with carbon dioxide contrast injection (A) shows internal enhancement of the hemorrhagic abscess (red arrow), confirming the hepatoduodenal fistula (blue arrow) (B).

The catheter was removed over a wire, and an 11 French vascular sheath was advanced. A selective catheter and guidewire were utilized to catheterize the fistula. Carbon dioxide angiography was injected through the vascular sheath without identifiable venous or arterial vessel communication (Figure 4).

**Figure 5:**
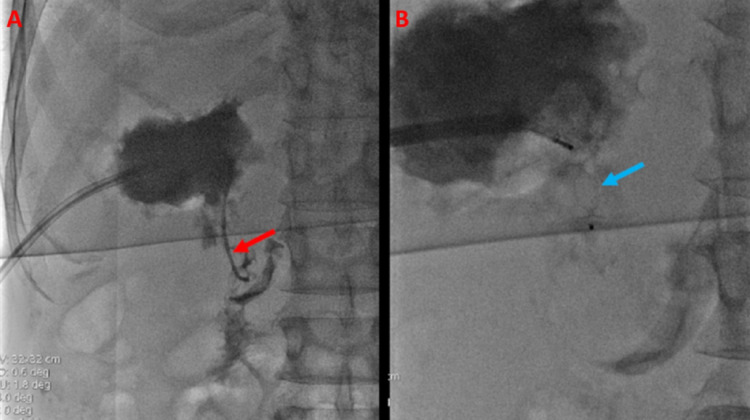
Deployment of an Amplatzer Vascular Plug Into the Hepatoduodenal Fistula An intra-procedural image (A) demonstrates the guidewire positioned within the hepatoduodenal fistula (red arrow). A subsequent intra-procedural image (B) demonstrates the deployment of an eight-millimeter Amplatzer® vascular plug (St. Jude Medical, Plymouth, MN) within the hepatoduodenal fistula (blue arrow).

Repeat abscessogram was performed with iodinated contrast, and again, no arteries or veins were identified. The decision was made to place an 8 mm Amplatzer® vascular plug (St. Jude Medical, Plymouth, MN) device within the fistula (Figure 5).

**Figure 6:**
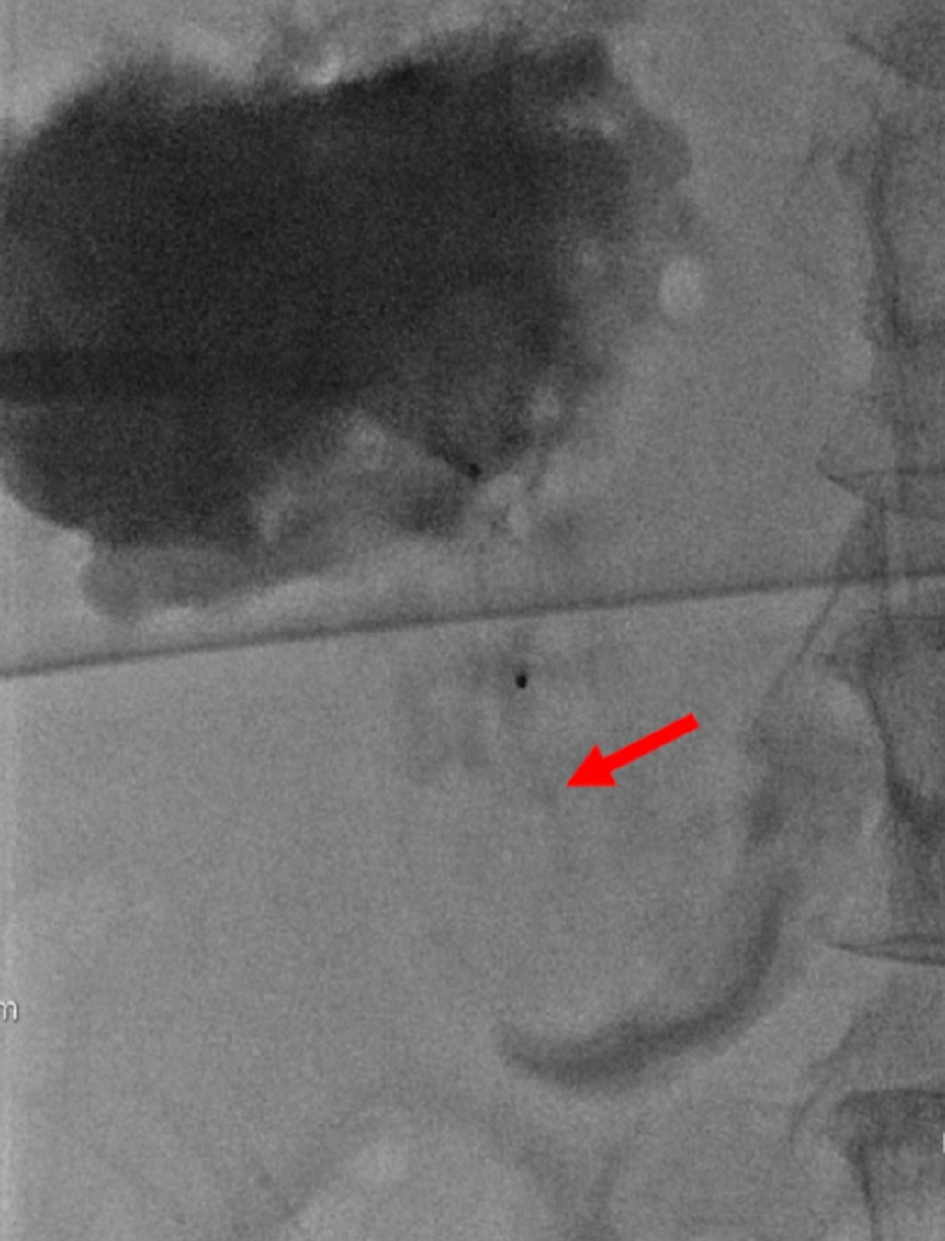
Post Amplatzer Vascular Plug Deployment Abscessogram Demonstrating Complete Occlusion of the Hepatoduodenal Fistula Following deployment of the Amplatzer® vascular plug (St. Jude Medical, Plymouth, MN), contrast injection through the percutaneous drain demonstrates filling of the hemorrhagic abscess and complete occlusion of the hepatoduodenal fistula (red arrow).

A repeat contrast injection demonstrated no further drainage into the duodenum (Figure 6).

**Figure 7:**
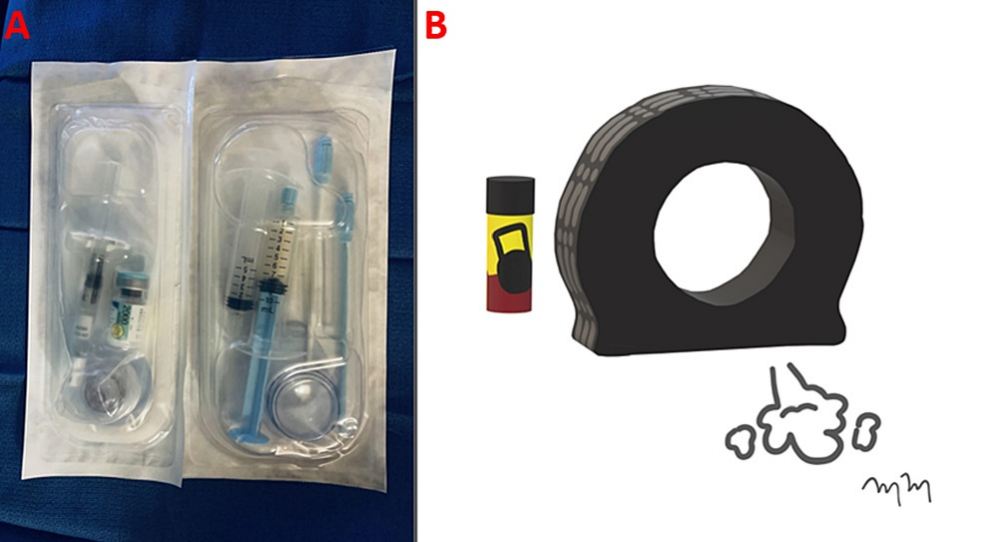
Artist Rendition of Aerosol Tire Inflator Analogous to SURGIFLO SURGIFLO® (Ethicon, Somerville, NJ) hemostatic matrix gelatin and thrombin kit (A) are shown, which was utilized for injection into the hemorrhagic intrahepatic abscess. An artist rendition image shows the actively deflating tire with an aerosol tire inflator, representing the injectable hemostatic matrix (B).

Subsequently, SURGIFLO® (Ethicon, Somerville, NJ) hemostatic matrix gelatin was mixed with contrast and administered in the abscess cavity to promote coagulation and prevent further hemorrhage (Figure 7).

**Figure 8:**
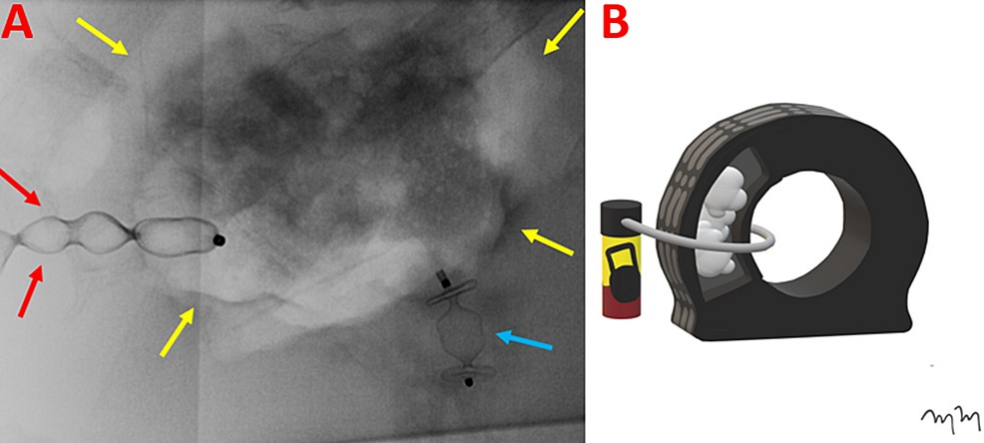
Artist Rendition of a Repaired Tire Analogous to Hemostasis of Hemorrhagic Intrahepatic Abscess Removal of the pigtail catheter with the deployment of a 16-millimeter Amplatzer® vascular plug (St. Jude Medical, Plymouth, MN) (A) within the tract of the percutaneous drain is shown (red arrows). Outlined carbon dioxide contrast (yellow arrows) following SURGIFLO® (Ethicon, Somerville, NJ) injection into the hemorrhagic intrahepatic abscess demonstrates complete occlusion. An eight-millimeter Amplatzer® vascular plug is again seen occluding the hepatoduodenal fistula (blue arrow). An artist rendition image (B) shows the insufflation of the tire with aerosol tire inflator matrix and complete repair of the air leak. This is analogous to the hemostasis of the hemorrhagic abscess by injection of the hemostatic matrix gelatin into the fistulous tracts with Amplatzer® vascular plugs.

This technique is similar to the commercially available aerosol tire repair in sealing any small air leaks within an automobile tire (Figure 8).

**Figure 9:**
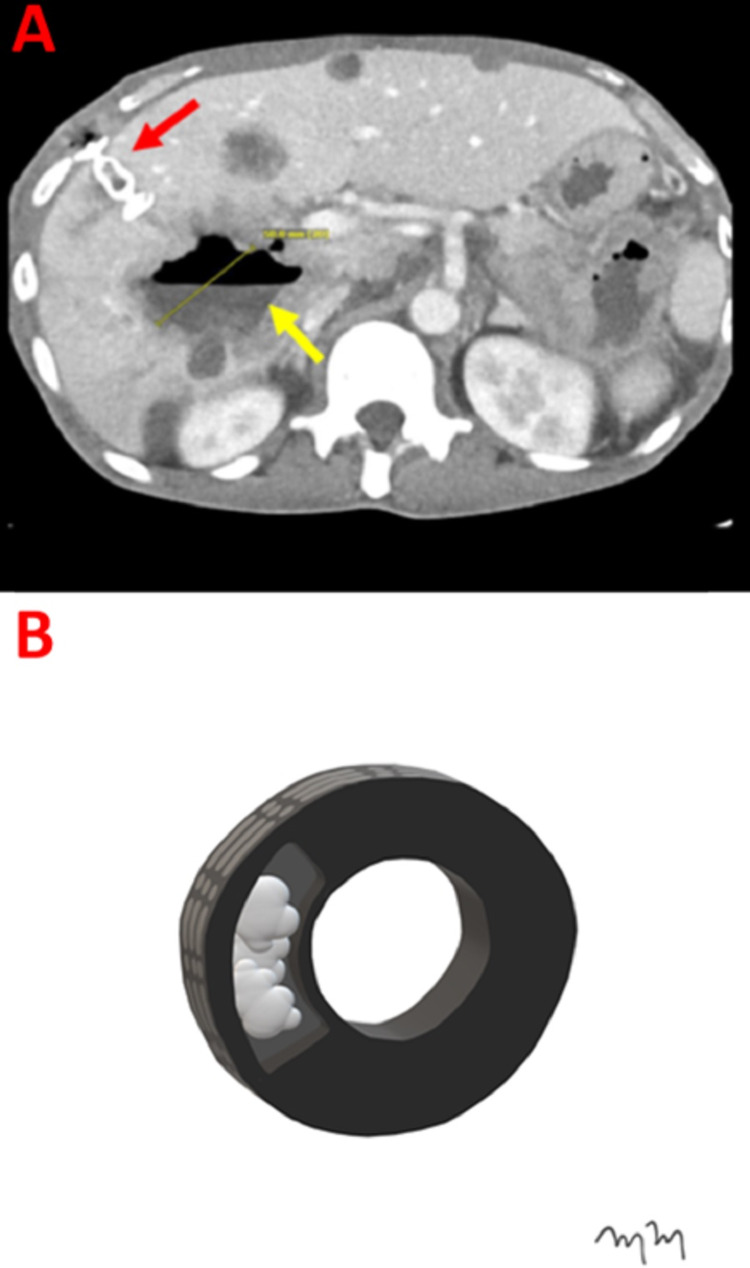
Artist Rendition of a Repaired Tire With Aerosol Tire Repair Analogous to Hemostatic Intrahepatic Abscess A post-procedural axial multiphasic computed tomography at the level of the inferior right hepatic lobe is shown (A). There is a significant interval decrease in size of the hemorrhagic intrahepatic abscess and expected air-fluid level within the mass (yellow arrow) related to the SURGIFLO® (Ethicon, Somerville, NJ) hemostatic matrix gelatin injection. There is no evidence of internal hemorrhage. The Amplatzer® vascular plug (St. Jude Medical, Plymouth, MN) within the previous percutaneous drain is in an unchanged position (red arrow) and without hemorrhagic extravasation. An artist rendition image (B) shows a functionally inflated tire with the aerosol tire repair matrix and complete repair of the air leak, analogous to the hemostatic intrahepatic abscess.

A 16 mm Amplatzer® vascular plug was placed through the existing drain access to occlude the tract (Figure 9).

**Figure 10:**
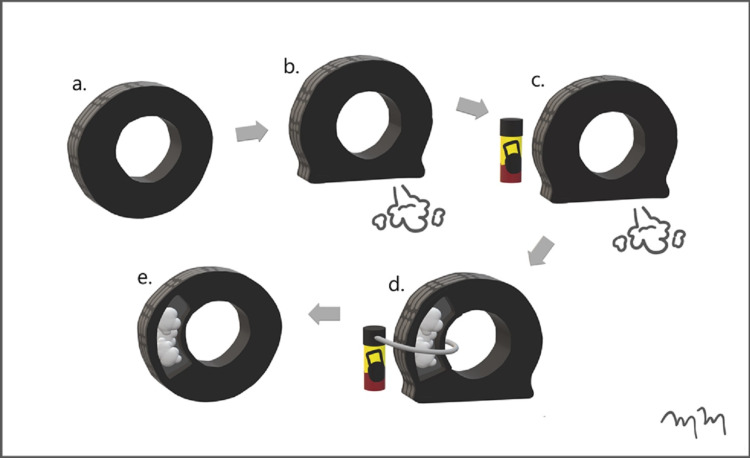
Artist Rendition Summary Diagram Demonstrating the Aerosol Tire Repair Analogy Relating the Repair of a Fistulized Intrahepatic Abscess An artist rendition summary flow diagram shows a functional tire (a) that develops an air leak (b). Aerosol tire repair matrix (c) is used to insufflate the leaking tire (d). Following insufflation with the aerosolized repair matrix, the tire is functional and without any air leak (e). The artist rendition is analogous to the hepatoduodenal fistula formed by an intrahepatic abscess, which leads to hemorrhage. The intrahepatic abscess is filled with percutaneously injected hemostatic matrix gelatin and thrombin material and is subsequently plugged with vascular occlusion devices, achieving hemostasis.

The patient tolerated the procedure well without any complications and was discharged home the same day. A follow-up outpatient CT of the abdomen two weeks later showed a decrease in the size of the abscess cavity without any evidence of further hemorrhage. Both of the Amplatzer® plugs remained in appropriate position. The patient's vital signs were stable and had improved hemoglobin and hematocrit values, negating any need for further percutaneous intervention. The following is a summary diagram of the aerosol tire repair analogy as it relates to the hemostasis of a fistulized intrahepatic abscess (Figure 10).

## Discussion

Although the formation of a hepatoduodenal fistula from a necrotic hepatic abscess is a rare occurrence, it is important to employ minimally invasive techniques to ensure resolution, and to decrease the risks of post-procedural complications [2-3]. The previously described novel percutaneous method for hemostasis of an intrahepatic abscess resulted in the complete resolution of the patient's symptoms. 

Hemostatic matrix gelatins, such as SURGIFLO®, are an efficacious and safe agent used to help stabilize bleeding in a wide variety of procedures during cranial, spinal, and cardiothoracic surgery [9-10]. These hemostatic agents have even been used specifically for the occlusion of femoral fistulas during percutaneous transfemoral aortic valve replacements [11]. In addition to the hemostatic matrix gelatin, two Amplatzer® vascular plugs were used; one plug within the fistulous tract and the other within the access exit tract. The Amplatzer® vascular plug is an embolic device that has proven to be a safe and effective tool for arterial embolization, venous occlusion, and treatment of abnormal vascular fistulas [12]. These devices were used in the preceding case in an off-label fashion by providing a functional barrier to prevent further hemorrhage and possible exsanguination.

The combination of the SURGIFLO® and Amplatzer® vascular plugs employed for the repair of the necrotic intrahepatic abscess prevented further invasive surgical management and sustained the patient's life. The high morbidity and mortality of the patient's metastatic squamous cell carcinoma remained the central concern for choosing to perform percutaneous hemostasis as previously described, over extensive surgical management. With the cessation of active acute hemorrhage from the hepatic drain, the patient’s anemia resolved, leading to return to patient baseline and later discharge. Additionally, the occlusion of the abscess drainage site with the Amplazter® vascular plug allowed for removal of the hepatic drain and relief of any unnecessary discomfort associated with a percutaneous drain.

## Conclusions

Due to the low incidence and lack of clinically validated management, the necessity remains for the definitive percutaneous treatment of hemorrhagic intrahepatic lesions as described in the preceding case. This case demonstrates a successfully treated percutaneous method for the management of a necrotic and hemorrhagic intrahepatic neoplasia with secondary hepatoduodenal fistula. Alternatively, invasive surgical management of fistulized intrahepatic necrotic neoplasias includes a greater risk for postoperative complications, including patient death. The novel application of injected hemostatic matrix gelatin and thrombin material into a fistulized intrahepatic mass with the occlusion of vascular plugs is analogous to the repair of a leaking automobile tire with a commercially available aerosol tire repair matrix.
